# Abundant Allelochemicals and the Inhibitory Mechanism of the Phenolic Acids in Water Dropwort for the Control of *Microcystis aeruginosa* Blooms

**DOI:** 10.3390/plants10122653

**Published:** 2021-12-02

**Authors:** Jixiang Liu, Yajun Chang, Linhe Sun, Fengfeng Du, Jian Cui, Xiaojing Liu, Naiwei Li, Wei Wang, Jinfeng Li, Dongrui Yao

**Affiliations:** 1 Jiangsu Key Laboratory for the Research and Utilization of Plant Resources, Institute of Botany, Jiangsu Province and Chinese Academy of Sciences (Nanjing Botanical Garden Mem. Sun Yat-Sen), Nanjing 210014, China; ljx891654338@163.com (J.L.); linhesun@cnbg.net (L.S.); duffyx@163.com (F.D.); jcui@cnbg.net (J.C.); liuxiaojingcau@126.com (X.L.); njbgkg@163.com (N.L.); izjwang@163.com (W.W.); lijinfeng1209@163.com (J.L.); 2 Jiangsu Engineering Research Center of Aquatic Plant Resources and Water Environment Remediation, Nanjing 210014, China

**Keywords:** *Microcystis aeruginosa*, biological control, water dropwort, allelopathic metabolite, phenolic acids, cellular structure, photosynthesis, antioxidant enzyme

## Abstract

In recent years, with the frequent global occurrence of harmful algal blooms, the use of plant allelopathy to control algal blooms has attracted special and wide attention. This study validates the possibility of turning water dropwort into a biological resource to inhibit the growth of harmful *Microcystis aeruginosa* blooms via allelopathy. The results revealed that there were 33 types of allelopathic compounds in the water dropwort culture water, of which 15 were phenolic acids. Regarding water dropwort itself, 18 phenolic acids were discovered in all the organs of water dropwort via a targeted metabolomics analysis; they were found to be mainly synthesized in the leaves and then transported to the roots and then ultimately released into culture water where they inhibited *M. aeruginosa* growth. Next, three types of phenolic acids synthesized in water dropwort, i.e., benzoic, salicylic, and ferulic acids, were selected to clarify their inhibitory effects on the growth of *M. aeruginosa* and their mechanism(s) of action. It was found that the inhibitory effect of phenolic acids on the growth of *M. aeruginosa* increased with the increase of the exposure concentration, although the algae cells were more sensitive to benzoic acid than to salicylic and ferulic acids. Further study indicated that the inhibitory effects of the three phenolic acids on the growth of *M. aeruginosa* were largely due to the simultaneous action of reducing the number of cells, damaging the integrity of the cell membrane, inhibiting chlorophyll a (Chl-a) synthesis, decreasing the values of *F*_0_ and *F*_v_*/F*_m_, and increasing the activity of the antioxidant enzymes (SOD, POD, and CAT) of *M. aeruginosa*. Thus, the results of this study indicate that both culture water including the rich allelochemicals in water dropwort and biological algae inhibitors made from water dropwort could be used to control the growth of noxious algae in the future.

## 1. Introduction

Harmful algal blooms formed in eutrophic water by the outbreak and growth of cyanobacteria can reduce biological diversity, destroy aquatic ecosystems, and endanger human health and safety [1,2,3]. As one of the main species of harmful algal blooms, *Microcystis aeruginosa* is widely distributed in freshwater systems worldwide [4,5,6]. Various methods have been developed to control *M. aeruginosa* blooms, including the use of ultrasound [7], chemical oxidants [8], photocatalysis [9], modified biochar [10], algaecides [11], biological control agents [12], and bioflocculants [13], among others. Although these techniques can effectively control algal blooms under certain circumstances, their disadvantages and limitations, including their high cost, complex operation, and ease of causing secondary water contamination [14], are evident. Thus, the development of efficient, economic, and environmentally friendly technologies for the prevention and inhibition of the growth of *M. aeruginosa* to control algal blooms is of primary importance, and has become a research hotspot in the fields of water resources protection and ecosystem management.

Allelopathy is defined as any direct or indirect beneficial or harmful effects of an organism on others via the release of chemical substances (known as allelochemicals) into the environment [15,16]. Many macrophytes have been reported as having strong allelopathic activity against various algae and can inhibit the growth of harmful algal blooms [17,18]. Moreover, allelochemicals released by macrophytes could be used as natural biocides for ecological security to control various algae in rivers, lakes, and seas due to their characteristics including biodegradability, a natural origin, and less pollution than traditional algaecides [19,20]. In recent years, with the frequent occurrence of harmful algal blooms globally, the use of plant allelopathy to control algal blooms has attracted unique and widespread attention [19,21,22]. More than 20 types of plant allelochemicals have been discovered, including terpenes, fatty acids, phenolic acids, etc. [20,23]. Previous studies have also demonstrated that specific chemicals released by plants are the main reason for the inhibition of algal growth by plants via the destruction of the cellular structure and the suppression of physiological responses like photosynthesis [17,18]. However, the allelochemicals released from plants vary among different species and tissues [24,25]. Features like the ecological safety and allelopathic efficiency of each macrophyte species must be estimated and considered before allelopathy can be applied in aquatic systems. In addition, to further investigate the allelopathic mechanism of plants against harmful algal blooms, the metabolites released and synthesized from plants should be identified and quantified [18,26].

Water dropwort (*Oenanthe javanica* (Blume) DC), an aquatic plant distributed in East Asian countries, is widely cultivated in China as a common vegetable with high nutritional value and medicinal function [27,28,29]. It is characterized by the environmental adaption features of fast growth, high nutrient absorption, strong reproductive capacity, being evergreen in all seasons, and easy management [30]. Therefore, it has been recognized as an ideal material for wastewater phytoremediation in China [31,32]. However, after wastewater bioremediation human consumption of water dropwort may present safety risks. As the extract of water dropwort has proven to have an allelopathic effect on algae [33], turning water dropwort into a biological resource after bioremediation to inhibit the growth of harmful *M. aeruginosa* should be considered. Therefore, to use water dropwort as a biological resource to control *M. aeruginosa* blooms, this research included the following: (i) the chemical class, contents, and release possibility of allelochemicals in water dropwort were analyzed using LC-ESI-MS-based metabolomics technology; (ii) the inhibitory effects of typical allelochemicals present in all organs of water dropwort on the growth of *M. aeruginosa* under different concentrations were examined by measuring the algal density; (iii) the inhibitory mechanism of phenolic acids in all organs of water dropwort on the physiological activity of *M. aeruginosa* was elucidated by observing the cell morphology and measuring both photosynthesis and antioxidant enzyme activity. The results provide a theoretical basis for the use of water dropwort to control algal blooms and for the development of an environmentally friendly, low-cost, and efficient cyanobacteria control agent.

## 2. Results

### 2.1. Inhibitory Effect of the Water Dropwort Culture Water on M. aeruginosa and the Identification of Metabolomics

**Figure 1:**
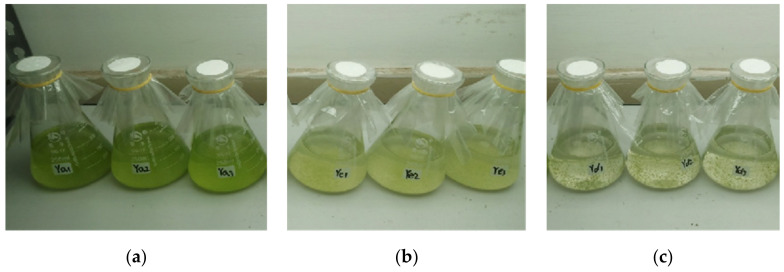
The inhibitory effect of the water dropwort culture water on the growth of *M. aeruginosa* on day 7. (**a**) 0 mL, (**b**) 25 mL, and (**c**) 50 mL of culture water were added to Erlenmeyer flasks containing 100 mL of *M. aeruginosa*, and the total volume of the culture solution in each group was maintained at 200 mL by replenishing sterile water. The final cell concentration was 2 × 10^7^ cells/mL. Each treatment included three parallel samples.

Figure 1 presents the inhibitory effects of different proportions of culture water from water dropwort planting on the growth of *M. aeruginosa*. The *M. aeruginosa* in the control group grew rapidly during the experiment, and the cell density increased from the initial value of 2 × 10^7^ cells/mL to 18.6 × 10^8^ cells/mL on day 7 (Figure 1a). In the 25-mL culture water treatment group, although the *M. aeruginosa* maintained a stable growth state, not only was the cell density on day 7 (10.9 × 10^7^ cells/mL) significantly lower than that of the control group, but the culture color also changed to yellow from normal green (Figure 1b), thereby indicating that *M. aeruginosa* was in a sub-healthy state and signifying the inhibition of the growth of algal cells. In the 50-mL culture water treatment group, the cell density significantly decreased and the cells became flocculent, and settled to the bottom on day 7 (Figure 1c); this indicates that *M. aeruginosa* could not survive in this amount of culture water. These findings demonstrate that the water dropwort culture water could inhibit or prevent the growth of *M. aeruginosa*.

**Figure 2:**
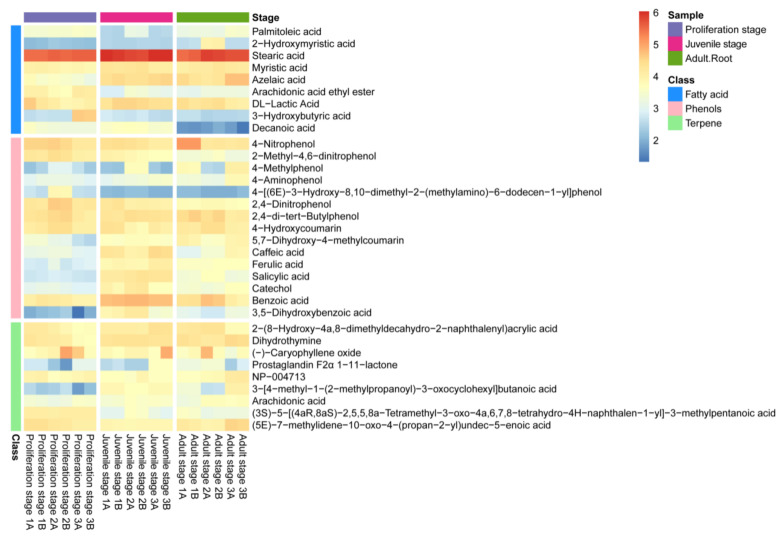
The heatmap of the contents of allelopathic compounds in the culture water of water dropwort at different growth stages. The bricks represent the log10A abundance of each compound in each sample. The histogram of different colors on the left represents fatty acids (blue), phenolic acids (pink), and terpenes (green). The histogram of different colors in the top-right corner represents samples from different growth stages of water dropwort.

The non-targeted metabolomics analysis of the water dropwort culture water was further carried out to identify the allelochemicals present in it (Figure 2). The results revealed the detection of a total of 306 metabolites in the culture water, among which 33, including fatty acids, phenolic acids and terpenes, have been proven to have a potential allelopathic function [34]. The heatmap in Figure 2 presents the relative abundances of these allelopathic compounds in the culture water, of which 15 were phenolic acids, nine were fatty acids, and nine were terpenes. Moreover, most of the phenolic acids in the culture water had a high abundance (Figure 2); the phenolic acids included 4-nitrophenol, 2-methyl-4,6-dinitrophenol, 4-methylphenol, 2,4-dinitrophenol, 2,4-di-t*ert*-butylphenol, caffeic acid, ferulic acid, 4-aminophenol, 5,7-dihydroxy-4-methylcoumarin, 4-hydroxycoumarin, salicylic acid, 4-[(6*E*)-3-hydroxy-8,10-dimethyl-2-(methylamino)-6-dodecen-1-yl]-phenol, catechol, benzoic acid, and 3,5−dihydroxybenzoic acid. Therefore, the water dropwort culture water was not only rich in allelochemicals at different development stages, but was also particularly abundant in phenolic acids. Thus, it can be inferred that these phenolic acids might play an important role in the allelopathy of the water dropwort culture water.

**Figure 3:**
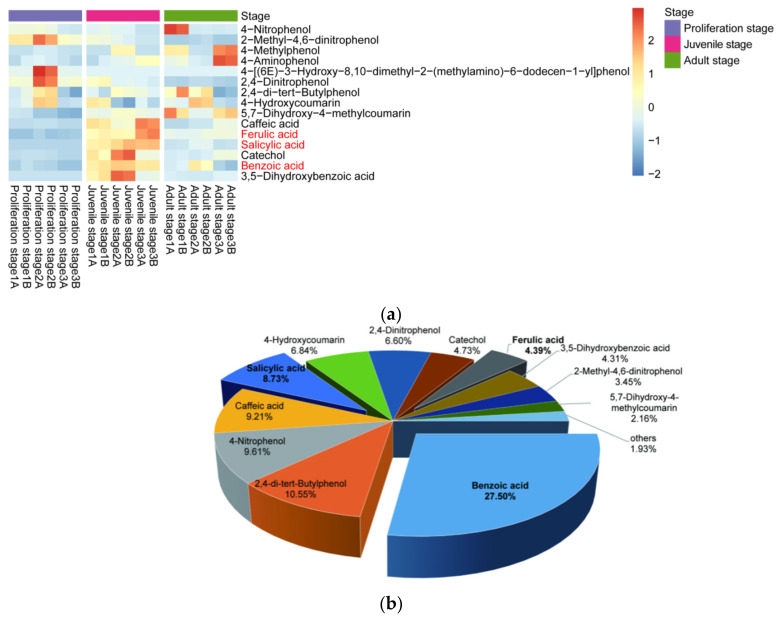
Relative abundances of allelopathic phenols in the culture water. (**a**) The heatmap of the contents of allelopathic phenols in the culture water of water dropwort at different growth stages. The bricks represent the relative abundance of each compound in each sample. The values are centered and scaled in the row direction to show the content differences of each compound more clearly among different stages. Compound names in red text signify the compounds further investigated in this study. (**b**) The relative abundances of allelopathy phenols in the juvenile-stage culture water.

**Figure 4:**
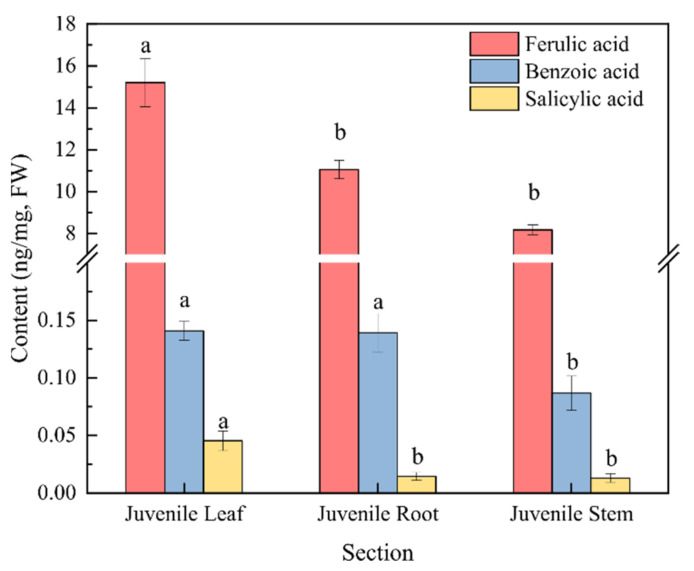
The contents of three phenolic acids in the targeted metabolomics analysis of water dropwort. There are three repetitions in each group. The data are reported as the mean ± standard error. Lowercase letters indicate a significant difference between the same phenolic acid in different organs.

Moreover, the content of phenolic acids in the culture water planted with juvenile-stage water dropwort was greater than the contents in both the proliferation-stage and adult-stage culture water (Figure 3a). The relative abundances of benzoic, salicylic, and ferulic acids at the juvenile stage were relatively high and characterized by good repeatability, and their average relative abundances were 27.5%, 8.73%, and 4.39%, respectively (Figure 3b). To further confirm whether the phenolic acids in the culture water were synthesized and released by the water dropwort itself, targeted metabolomics analysis for phenolic acids was performed for the entire water dropwort plant at the juvenile stage (Figure 4). Although the contents of phenolic acids in different tissues were significantly different, the 15 phenolic acids, including ferulic, benzoic, and salicylic acids, were detected in all three organs of the roots, stems, and leaves of the juvenile water dropwort (Supplementary Table S1). From highest to lowest, the contents of the ferulic, benzoic, and salicylic acids in different tissues were those in the leaves, roots, and stems, and the content of ferulic acid in the water dropwort leaves was as high as 15.20 ± 1.15 ng/mg (Figure 4). These results suggest that allelopathic phenolic acids were abundant in the water dropwort, and they were involved in the inhibition of the growth of *M. aeruginosa* via release into the culture water.

### 2.2. Effects of Different Phenolic Acids on the Growth of M. aeruginosa

Various phenolic acids contained in both the culture water and extracted samples of water dropwort were identified by both qualitative and qualitative analysis. Moreover, the contents of benzoic, salicylic, and ferulic acids in the water dropwort were much higher than the contents of other phenolic acids. Thus, the inhibitory effects of these phenolic acids on the growth of *M. aeruginosa* were studied by respectively adding them to the *M. aeruginosa* to further investigate the allelopathic mechanism. Considering the universal discovery characteristics of “low to promote and high to suppress” regarding the allelopathic inhibition of harmful algal blooms [35,36], pre-tests were first carried out to identify the inhibitory dose of phenolic acids for *M. aeruginosa* (Supplementary Figure S1). Compared with the control group, the growth of *M. aeruginosa* was initially inhibited by 20 mg/L of benzoic acid and salicylic acid, respectively. Moreover, when the concentrations of benzoic acid and salicylic acid respectively reached 40 mg/L, the growth of *M. aeruginosa* cells was completely prevented. However, 20 mg/L of ferulic acid significantly promoted the growth of *M. aeruginosa*, while 60 mg/L of ferulic acid completely prevented the growth. Thus, the concentrations of benzoic acid and salicylic acid were both set to 25, 30, 35, and 40 mg/L, and the concentrations of ferulic acid were in the range of 30 to 60 mg/L.

**Figure 5:**
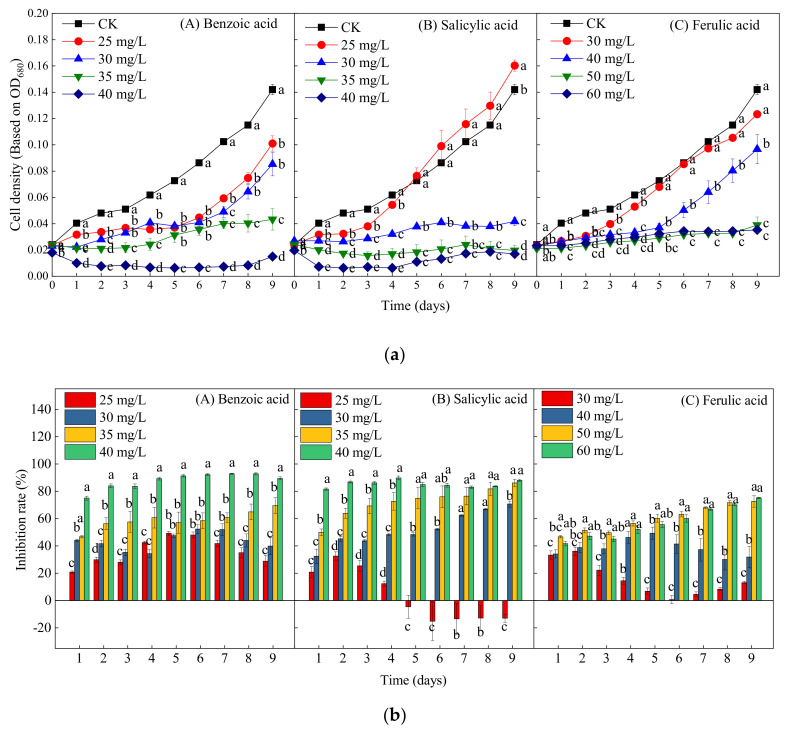
The effects of different concentrations of ferulic, salicylic, and benzoic acids on the growth of *M. aeruginosa*. (**a**) The change in the cell density of *M. aeruginosa* with treatment by different phenolic acids. (**b**) The inhibition rate of *M. aeruginosa* with treatment by different phenolic acids. There are three repetitions in each group. The data are reported as the mean ± standard error. Lowercase letters of different treatments at the same time indicate significant differences.

Figure 5 presents the growth curves and inhibition efficiency of *M. aeruginosa* treated by different concentrations of the three phenolic acids. In contrast to the control group, all four concentrations (25, 30, 35, and 40 mg/L) of benzoic acid significantly inhibited the growth of *M. aeruginosa* during the experiment (*p* < 0.05); the inhibition rates of these four concentrations on day 9 were respectively 28.87%, 39.93%, 69.51%, and 89.44%, thereby reflecting a dose-dependent inhibitory effect (Figure 5(bA)). Moreover, at the highest concentration of benzoic acid (40 mg/L), the decline in cell density began at the beginning of cultivation, indicating growth arrest and cell death (Figure 3a). At the lowest concentration of salicylic acid (25 mg/L), the algal growth was first suppressed and then promoted after 5 days, and the growth increased by 12.89% on day 9 as compared with the control (Figure 5a,b). With the increase of the concentration of salicylic acid to 30, 35, and 40 mg/L, the inhibition rates on day 9 respectively reached 70.63%, 86.13%, and 88.03% (Figure 5(bB)), thereby also reflecting obvious dose-dependent inhibitory effects. The ferulic acid concentration of 30 mg/L significantly inhibited the algal growth within 5 days, but, after day 5, there were no significant differences between the 30-mg/L ferulic acid treatment and the control. The cell density under the 40-mg/L ferulic acid treatment did not increase or decrease significantly within 6 days, which was then followed by a rapid growth trend, indicating growth recovery. Nevertheless, high concentrations of ferulic acid (50 and 60 mg/L) significantly inhibited the growth of *M. aeruginosa* during the entire experiment, with inhibition rates of 72.54% and 75.14%, respectively (Figure 5(bC)). In general, the inhibitory effects of the three phenolic acids on *M. aeruginosa* all increased with the increase of the exposure concentration. Furthermore, the inhibitory effects of benzoic and salicylic acids were significantly stronger than that of ferulic acid.

### 2.3. Effects of Different Phenolic Acids on the Cell Morphology of M. aeruginosa

**Figure 6:**
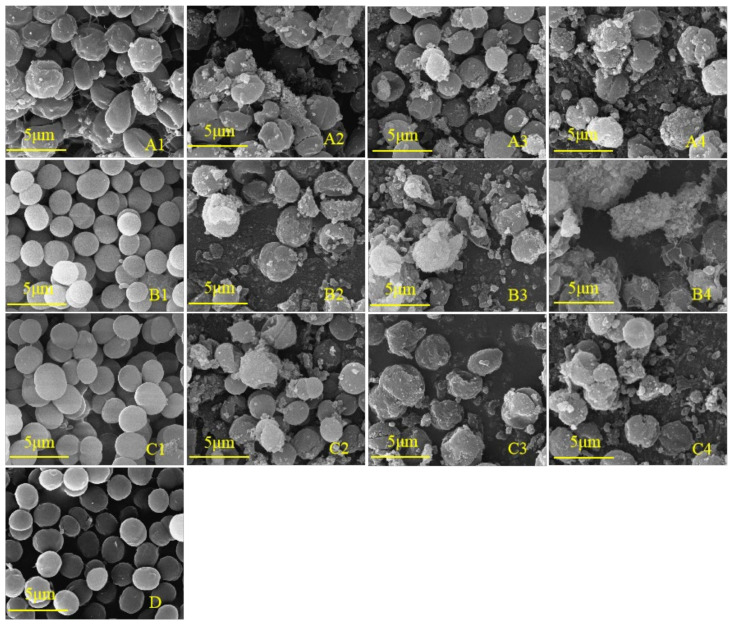
SEM images of *M. aeruginosa* treated with different concentrations of phenolic acid. (**A1**–**A4**): 25, 30, 35, and 40 mg/L benzoic acid treatment, respectively; (**B1**–**B4**): 25, 30, 35, and 40 mg/L salicylic acid treatment, respectively; (**C1**–**C4**): 30, 40, 50, and 60 mg/L ferulic acid treatment, respectively; (**D**): control of the BG-11 medium.

To further study whether the cell development of *M. aeruginosa* is inhibited by phenolic acids, the cell morphology was observed. Scanning electron microscopy (SEM) is often used as an intuitive and indispensable tool to estimate algal cell damage [37,38]. As shown in Figure 6D, the algal cells in the control group were distinct, round, and plump with a smooth exterior and complete morphology, and were split vigorously. The cell morphologies under the lowest concentrations of salicylic acid (25 mg/L) and ferulic acid (30 mg/L) were not obviously affected as compared with the control (Figure 6(B1,C1)). In contrast, the lowest concentration of benzoic acid (25 mg/L) that significantly inhibited the growth of *M. aeruginosa* caused the evident shrinking of the cell surface (Figure 6(A1)), suggesting that the algal cells were more sensitive to benzoic acid than to salicylic and ferulic acids. In contrast to the control, the number of cells per unit area was decreased, the number of inclusions on the cell surface was increased, and the morphology became irregular under the treatments of 30 mg/L benzoic and salicylic acids (Figure 6(A2,B2)), indicating the cracking of cells and plasma membrane lysis. These results demonstrate that with the increase of the concentration of the three phenolic acids, the number of cyanobacteria cells per unit area gradually decreased, while the extracellular flocculent aggregates and extracellular secretions increased (Figure 6).

### 2.4. Effects of Different Phenolic Acids on the Chl-a Content in M. aeruginosa

**Figure 7:**
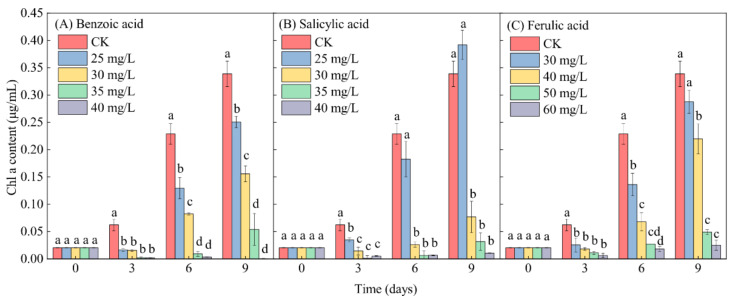
The effects of different concentrations of phenolic acids on the Chl-a content in *M. aeruginosa*. There are three repetitions in each group. The data are reported as the mean ± standard error. Lowercase letters at the same time indicate significant differences between different treatments.

In contrast to the control (CK), the Chl-a content in *M. aeruginosa* exhibited a lesser increase under relatively low-concentration phenolic acid treatments (≤30 mg/L for benzoic and salicylic acids, and ≤40 mg/L for ferulic acid) during the entire growth period of *M. aeruginosa*. The Chl-a content even exhibited a significant decrease under relatively high-concentration phenolic acid treatments (≥35 mg/L for benzoic acid and salicylic acid, and ≥50 mg/L for ferulic acid), with the exception of the 25-mg/L salicylic acid treatment, after 9 days of growth (Figure 7). This reflects the typical inhibitory effect of phenolic acids on the growth of *M. aeruginosa*. Under the same low-concentration conditions of 30 mg/L phenolic acid, the inhibitory effects of benzoic, salicylic, and ferulic acids on the increase of the Chl-a content in *M. aeruginosa* respectively reached 54.01%, 77.33%, and 15.00% on day 9. At high concentrations (≥35 mg/L) of benzoic and salicylic acids, the inhibitory effects of benzoic acid on the increase of the Chl-a content during the 9 days of *M. aeruginosa* growth respectively reached 84.09% and 99.88%, and those of salicylic acid respectively reached 90.64% and 96.90%. A similar inhibition to the increase of the Chl-a content in *M. aeruginosa* was detected under the high-concentration ferulic acid treatment (≥50 mg/L), and the inhibitory effect even reached 92.62% under the 60-mg/L ferulic acid treatment after 9 days of growth (Figure 7C). In summary, with the increase of the concentration of phenolic acids, the Chl-a content in *M. aeruginosa* in the same period decreased significantly. The extent of the decrease of the Chl-a content was found to be basically consistent with the analysis of algal growth inhibition, and was concentration-dependent. Moreover, high concentrations of all three phenolic acids significantly reduced the Chl-a content in *M. aeruginosa*.

### 2.5. Effects of Different Phenolic Acids on the Fluorescence Parameters of M. aeruginosa

**Figure 8:**
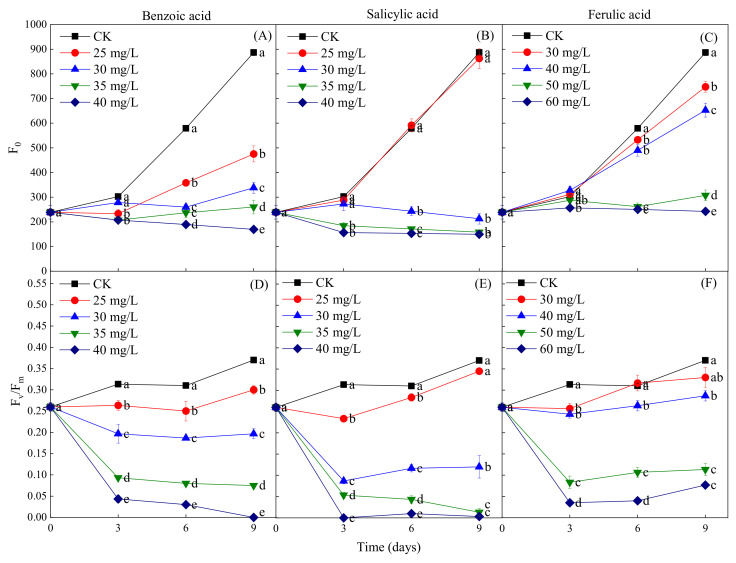
The fluorescence parameters *F*_0_ and *F*_v_*/F*_m_ of *M. aeruginosa* under different concentrations of phenolic acids. (**A**–**C**): The *F*_0_ of *M. aeruginosa* under benzoic acid, salicylic acid and ferulic acid treatments, respectively; (**D**–**F**): The *F*_v_*/F*_m_ of *M. aeruginosa* under benzoic acid, salicylic acid and ferulic acid treatments, respectively. There are three repetitions in each group. The data are reported as the mean ± standard error. Lowercase letters of different treatments at the same time indicate significant differences.

The fluorescence parameters *F*_0_ and *F*_v_*/F*_m_ were measured to determine the coefficient of light absorption and the efficiency of the photosynthetic reactions of *M. aeruginosa* in response to phenolic acids. The phenolic acid treatments were found to have a concentration-dependent inhibitory effect on the increase of *F*_0_ during the *M. aeruginosa* growth period, but the concentrations of different phenolic acids required for the inhibition were different. The inhibition of the increase of *F*_0_ was observed at all concentrations of benzoic acid (≥25 mg/L) during the entire growth period of *M. aeruginosa*, while significant inhibition was detected at the salicylic acid concentration of ≥30 mg/L after 3 days of growth and of the ferulic acid concentration of ≥30 mg/L after 6 days of growth (Figure 8A–C). This indicates that the *F*_0_ inhibition intensity of the three phenolic acids, from greatest to least, was benzoic acid > salicylic acid > ferulic acid. In addition, the results revealed that a certain concentration of phenolic acid treatments resulted in the decrease of *F*_v_*/F*_m_ in *M. aeruginosa* during its growth period; all concentrations of benzoic acid had this effect, as did salicylic acid at the concentration of ≥30 mg/L and ferulic acid at the concentration of ≥50 mg/L (Figure 8D–F). *F*_v_*/F*_m_ exhibited a sharp decline in the early stage of *M. aeruginosa* growth (the first 3 days) for all three phenolic acids at high concentrations; in contrast, in the later growth stage, *F*_v_*/F*_m_ decreased slowly under the benzoic acid treatment, and remained unchanged under the high-concentration salicylic and ferulic acid treatments. Specifically, *F*_v_*/F*_m_ declined to close to zero under the 40-mg/L benzoic and salicylic acid treatments on days 9 and 3 of *M. aeruginosa* growth, respectively, which indicates the stronger inhibitory effects of benzoic and salicylic acids than that of ferulic acid on the growth of *M. aeruginosa*. Furthermore, the fluorescence parameters *F*_0_ and *F*_v_*/F*_m_ were consistent with OD_680_ and the Chl-a content after exposure to phenolic acids.

### 2.6. Effects of Different Phenolic Acids on the Antioxidant Enzyme Activity of M. aeruginosa

**Figure 9:**
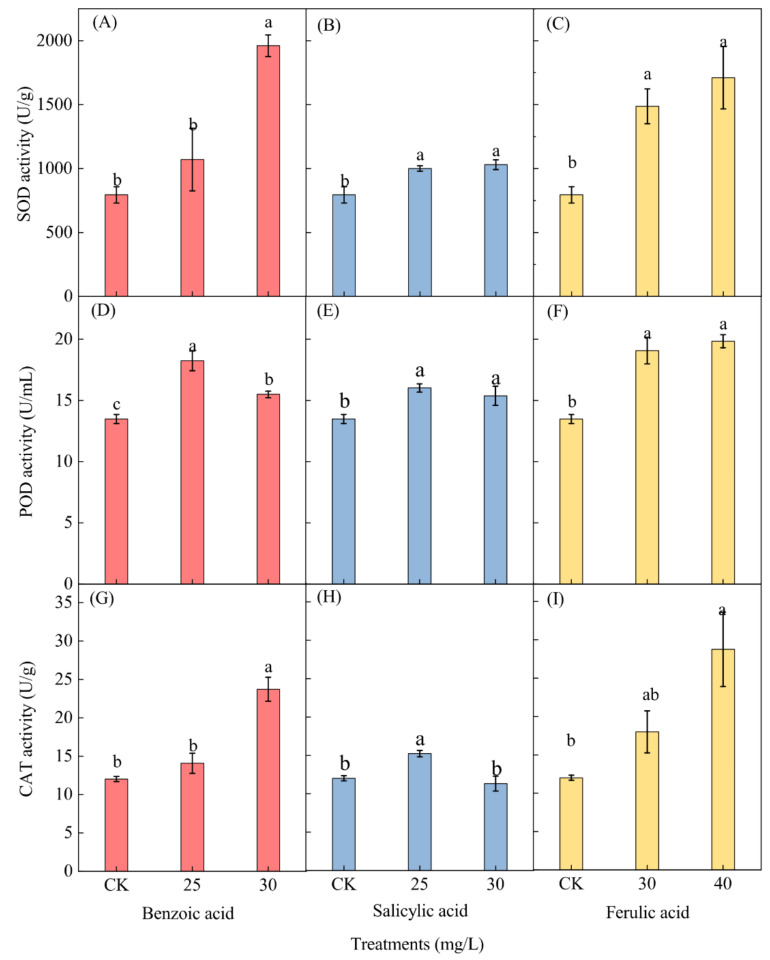
The effects of different concentrations of phenolic acid on the enzyme activities of *M. aeruginosa*. (**A**–**C**): The SOD activity of *M. aeruginosa* under benzoic acid, salicylic acid and ferulic acid treatments, respectively; (**D**–**F**): The POD activity of *M. aeruginosa* under benzoic acid, salicylic acid and ferulic acid treatments, respectively; (**G**–**I**): The CAT activity of *M. aeruginosa* under benzoic acid, salicylic acid and ferulic acid treatments, respectively. There are three repetitions in each group. The data are reported as the mean ± standard error. Lowercase letters of different treatments at the same time indicate significant differences.

The effects of the three different phenolic acids on the activity of antioxidant enzymes in *M. aeruginosa*, including superoxide dismutase (SOD), peroxidase (POD), and catalase (CAT), were measured at the end of the experiment. Only the enzyme activities of *M. aeruginosa* cells under low concentrations of phenolic acids (25 and 30 mg/L for benzoic and salicylic acids, and 30 and 40 mg/L for ferulic acid) were determined, while those under high concentrations of phenolic acids (≥35 mg/L for benzoic and salicylic acids, and ≥50 mg/L for ferulic acid) were omitted due to the insufficient collection of algal cell samples. A significant increase in the SOD activity of *M. aeruginosa* was observed for both concentrations of the salicylic and ferulic acid treatments and for the 30-mg/L benzoic acid treatment, but an insignificant increase was observed for the 25-mg/L benzoic acid treatment (Figure 9A–C). After phenolic acid treatment with 30 mg/L of benzoic and salicylic acids and 40 mg/L of ferulic acid, the SOD activity respectively increased 2.47-, 1.22-, and 2.12-fold as compared to the corresponding control.

Phenolic acid treatment induced a significant increase in the POD activity in *M. aeruginosa*. A higher activity level was detected under the 25 mg/L benzoic and salicylic acid treatments, as well as under the 40-mg/L ferulic acid treatment (Figure 9D–F). The POD activity in *M. aeruginosa* respectively increased 1.35- and 1.19-fold under the 25-mg/L benzoic and salicylic acid treatments as compared with the control, and increased 1.47-fold under the 40-mg/L ferulic acid treatment as compared to the control. The CAT activity displayed a similar trend as the SOD activity after phenolic acid treatment (Figure 9G–I). The enzyme activity in *M. aeruginosa* increased significantly under both the benzoic acid treatment of 30 mg/L and the ferulic acid treatment of 40 mg/L; 1.97- and 2.39-fold increases as compared with the control were respectively observed (Figure 9G,I). Furthermore, a significant increase (1.27-fold as compared to the control) of the CAT activity was observed under the 25-mg/L salicylic acid treatment (Figure 9H).

## 3. Discussion

In recent years, frequent outbreaks of harmful algal blooms in the eutrophic water of rivers, lakes, and seas have posed a significant threat to water resources [39]. *M. aeruginosa* in eutrophic water has been a popular research subject in recent decades due to its toxin-producing capability that threatens public health [40,41]. To date, many aquatic plants that can inhibit the growth of *M. aeruginosa* via the release of allelochemicals have been found, and they include submerged plants, emergent plants, and floating plants [42,43,44,45]. Nevertheless, different aquatic plants secrete different allelochemicals, and the types and quantities of allelochemicals in a plant vary within its different organs or development stages. Moreover, the synthesis and release pathways of allelochemicals in different plants are different. Nakai et al. [46,47,48] found that the allelochemicals secreted by *Myriophyllum spicatum* are mainly phenolic acids, including pyroic acid, gallic acid, and catechin. Gao [49] detected nine phenolic acids, namely benzoic acid, *p*-hydroxybenzoic acid, *p*-hydroxyphenylacetic acid, phthalic acid, *p*-hydroxybenzoic acid, vanillic acid, protocatechuic acid, ferulic acid, and caffeic acid, in the ethyl acetate component of *Vallasneria asiatica*. Hu [50] found that the allelopathic algae inhibitory substances produced by *Potamogeton malaianus* are mainly fatty acids. Huang [51] isolated more than 40 compounds from aqueous extracts of *Malayan cabbage*, *Hydrilla verticillata*, and *Vallaseria asiatica*, and found that most of them are fatty acids. Li [52] found that the allelochemicals secreted and released by *Phragmites communis* are 2-methyl acetoacetate. Furthermore, studies have shown that the allelopathic substances in *Eichhornia crassipes* that inhibit the growth of algae are mainly produced by the root [53], the algal inhibitory substances in *Acorus calamus* are mainly secreted and released by the root and rhizome, and the allelopathic substances in *Potamogeton maackianus* are mainly released by the stem and leaf [54]. In addition, some researchers have compared the inhibitory effects of the extracts from different parts of *Phragmites communis* on the growth of *Microcystis aeruginosa*, and found that the allelochemicals released from the roots have stronger inhibitory effects [55].

In the present study, 306 metabolites were detected in the culture water of water dropwort at different development stages. Of the 306 metabolites, including fatty acids, phenolic acids, and terpenes, 33 have been proven to have a potential allelopathic function [34], and the others either have no allelopathic function, or their corresponding allelopathic function is not clear. Regarding the 33 metabolites with an allelopathic function, almost half of them (15) were found to be phenolic acids, while the other 18 metabolites were either fatty acids (nine) or terpenes (nine). These results suggest that the culture water of water dropwort at different development stages was rich in allelochemicals, and was especially abundant in phenolic acids. To further confirm the components and secretory tissues of phenolic acids in the water dropwort culture water, in the present study, 18 phenolic acids, including caffeic acid, ferulic acid, and benzoic acid, were first identified in all organs of the water dropwort via targeted metabolomics analysis. Furthermore, the contents of phenolic acid in the different tissues of the water dropwort, from highest to lowest, were found to be those in the leaves, roots, and stems; this indicates that the phenolic acids in water dropwort are mainly synthesized in the leaves, and are then transported to the roots. These findings suggest that water dropwort has a significant ability to release phenolic acids into its growth environment via its root system, and the released phenolic acids are either relatively highly concentrated or accumulated within a certain period of time. Because phenolic acids have been widely used as allelochemicals to inhibit harmful algal growth [19,56,57,58], two promising approaches can be used to control the growth of noxious algae, namely: (1) utilizing water dropwort culture water, which is rich in allelochemicals, or (2) extracting phenolic acids from the leaves and roots of water dropwort to develop them into biological algae inhibitors.

To further clarify the inhibitory effects of different phenolic acids on *M. aeruginosa*, three types of phenolic acids synthesized in water dropwort, i.e., benzoic, salicylic, and ferulic acids, were selected for further investigation in the present study. The four concentrations (25, 30, 35, and 40 mg/L) of benzoic acid treatment were found to significantly inhibit the growth of *M. aeruginosa* during the entire experiment (*p* < 0.05), and the inhibitory effect was found to be concentration or dose-dependent. The cell density (OD_680_ value) in the *M. aeruginosa* culture solution remained below the initial inoculation value of 0.02 at the benzoic acid concentration of 40 mg/L, which indicated the growth arrest and cell death of *M. aeruginosa* in the culture solution (Figure 3A). Although the growth of *M. aeruginosa* was inhibited at the beginning stage under the low-concentration salicylic acid treatment (≤25 mg/L), it was significantly promoted in the later growth stage. The reason for the promotion of the growth of *M. aeruginosa* may have been the increase of the permeability of the algal cell membrane at the low concentration of salicylic acid, which made it easier for *M. aeruginosa* to absorb nutrients in the culture medium [59]. This promotion effect did not exist at salicylic acid concentrations greater than 25 mg/L. Conversely, the dose-dependent inhibitory effect was very prominent. Similarly, high concentrations of ferulic acid (≥50 mg/L) were also found to significantly inhibit the growth of *M. aeruginosa* during the entire experiment. The acceptable ranges of the concentrations of the three phenolic acids were different due to their distinct chemical structures. When the concentration of added phenolic acid exceeded the acceptable concentration range of algae, the cell membrane, photosynthetic, and/or antioxidant enzyme systems of the algae were damaged, and the degree of damage increased with the increase of the concentration of the treatment acids [60].

Changes in the cell morphology, photosynthesis parameters, and enzymatic activity of *M. aeruginosa* were investigated during 9 days of successive exposure to different doses of benzoic, salicylic, and ferulic acids to confirm the inhibitory effects of these phenolic acids. SEM was used to estimate the algal cell damage [37,38], and it was found that the algae cells under low concentrations of salicylic acid (≤25 mg/L) and ferulic acid (≤30 mg/L) exhibited no obvious distortion or morphological changes as compared with the control; this indicates that the morphological or structural integrity of the cells was not substantially affected under these conditions. As the concentrations of both phenolic acids increased, the number of algae cells per unit field of view under the microscope obviously decreased, and the extracellular flocculent aggregates and dead-cell lysis increased. However, a low concentration of benzoic acid (25 mg/L) caused an apparent change in the cell morphology, i.e., the swelling and shrinking of the cell surface, and even breakage and the occurrence of holes in the membrane. These findings suggest that the algae cells were more sensitive to benzoic acid than to salicylic and ferulic acids. Moreover, when the dose of each of the three phenolic acids exceeded the acceptable range of algal cell growth, the cell membrane was damaged seriously and even irreversibly, leading to the ultimate disintegration of *M. aeruginosa*. Hence, a certain concentration of allelochemicals, namely the three phenolic acids considered in the presented study, can damage the cell morphology and membrane integrity, resulting in the inhibition of algal cell growth. Similar findings were also reported by Hua [19] and Meng [61], i.e., extracts of *Ailanthus altissima* and rice straw were found to cause the distinct alteration of the cell morphology, the rupture of the cell membrane, and the leakage of the inclusion of *M. aeruginosa*.

Allelochemical stress generally reduces photosynthesis in some plant species, including algae [20]. Photosynthesis is one of the most important metabolic processes for green plants and algae, and its efficiency is usually reflected by the content of Chl-a, which plays an important role in energy capture and transfer in the photosynthetic process [62,63,64,65]. Consequently, the Chl-a content has been used as a monitoring indicator of the potential photosynthetic ability of *M. aeruginosa* [19,66]. In the present study, high concentrations of phenolic acids (≥35 mg/L for benzoic and salicylic acids, and ≥50 mg/L for ferulic acid) caused a significant decrease of the Chl-a content in *M. aeruginosa* (Figure 7), and the decline in the levels of Chl-a exhibited dose-dependency. The reduction of Chl-a would further lead to the photosynthesis-related metabolic dysfunction of algal cells [67,68]. In addition, it was found that the reduction of the cell density was positively correlated with the decrease of the Chl-a content, implying that another important mechanism of the inhibitory effect of phenolic acids on the growth of *M. aeruginosa* might be their inhibition of Chl-a synthesis [69]. Furthermore, considering the powerful function of chlorophyll fluorescence technology in the field of algae physiological and ecological research, the fluorescence parameters *F*_0_ and *F*_v_*/F*_m_ were further measured to reveal the reasons for the inhibition of algae photosynthesis under the phenolic acid treatments. *F*_v_*/F*_m_, namely the maximum photochemical quantum yield of PSII, can be used to illustrate the potential photosynthetic capacity of *M. aeruginosa* [57], and it would be significantly declined under external environmental stress, thereby indicating the damage of PSII [70,71]. It was found that the decreases of the *F*_0_ and *F*_v_*/F*_m_ parameters of algal cells caused by adding phenolic acids were dose-dependent. Regarding the entire process, the change of *F*_v_*/F*_m_ exhibited a decreasing trend, but it maintained a relatively gentle decline after 3 days of early cultivation. This can be attributed to the self-protection mechanism of the few surviving algal cells [72]. However, *F*_v_*/F*_m_ declined to near zero at high concentrations of benzoic and salicylic acids after certain treatments for successive days, suggesting the serious damage of PSII. In addition, among the three different phenolic acids, the inhibitory effects of benzoic and salicylic acids on the algae PSII were significantly stronger than that of ferulic acid. These results were consistent with the findings of the changes in OD_680_ and the Chl-a content in algal cells exposed to phenolic acids. Hence, the inhibitory effects of the three phenolic acids on *M. aeruginosa* growth were largely due to their damage to the algae PSII.

Algal cells, like most plant cells, have a complex oxygen defense system to protect themselves against oxygen-derived free radicals, including the following three main enzymes: superoxide dismutase (SOD), which eliminates superoxide radicals (O_2_^−^) by transforming them into hydrogen peroxide or ordinary molecular oxygen, and catalase (CAT) and peroxidase (POD), which remove H_2_O_2_ by decomposing H_2_O_2_ into H_2_O and molecular oxygen [73,74,75]. When algal or plant cells encounter adverse environmental stress, their antioxidant enzyme systems will usually be activated to eliminate reactive oxygen species (ROS) and alleviate membrane lipid peroxidation damage [76]. In this study, the antioxidant enzyme activities of SOD, CAT, and POD in *M. aeruginosa* were found to have increased significantly after 9 days of exposure to low concentrations of phenolic acids, indicating that the algae could clear up or alleviate the damage of ROS induced by phenolic acid treatments via the upregulation of antioxidant enzyme activities. Furthermore, no significant upregulation, or even the downregulation, of the enzyme activities was observed under the benzoic and salicylic acid treatments of 30 mg/L as compared with the treatment of 25 mg/L, nor under the ferulic acid treatment of 40 mg/L as compared with the treatment of 30 mg/L. This implies that a relatively higher concentration of phenolic acids could lead to the decrease of cellular antioxidant levels. In this case, the induced ROS can easily bring about overproduction due to the decrease of the free radical scavenging capacity of the algae antioxidant enzyme system, thereby causing serious damage to the cells. This, in fact, could explain to a great extent the changes of the algal growth during treatment with different concentrations of phenolic acids. Consistent findings were also reported in the research of the changes of the antioxidant enzyme activities of *M. aeruginosa* via rice straw aqueous extract and quercetin [19,71]. Therefore, the effect on the antioxidant system of *M. aeruginosa* is also one of the main mechanisms by which phenolic acids inhibit algal growth.

## 4. Materials and Methods

### 4.1. Culture of M. aeruginosa

*M. aeruginosa* FACHB-524 was purchased from the Freshwater Algae Culture Collection of the Institution of Hydrobiology, the Chinese Academy of Sciences. *M. aeruginosa* was cultured in 500-mL sterilized flasks with BG-11 medium at a temperature of 25 °C and a light-dark regime of 12 h: 12 h (with 4000 lux of illumination) in the laboratory [77]. The *M. aeruginosa* in each flask was shaken twice per day, and was then transferred and inoculated once every 7 days to maintain cell viability. The formula of the BG-11 medium for *M. aeruginosa* used in the present study is exhibited in Table 1.

### 4.2. Preparation of Water Dropwort Culture Water

Evergreen water dropwort material is a local cultivar from Suqian, Jiangsu Province, China. The suitable growth and survival temperatures are in the respective ranges of 5–30 °C and (−15)–40 °C. Water dropwort with a fresh weight of 150 g at three developmental stages (proliferation, juvenile, and adult) was planted in beakers with 1 L of pure water to obtain the culture water. One week later, the water samples from three replicates for each stage were divided into 45-mL samples and freeze-dried, after which 500 μL of methanol:water solution (7:3 *v*/*v*, containing 0.1% formic acid) was added to for redissolution. Next, the samples were centrifuged at 4 °C at 13,680× *g* for 10 min and then freeze-dried again for storage at −20 °C. Finally, the frozen samples were dissolved in 200 μL of 30% acetonitrile (*v*/*v*) and transferred to insert-equipped vials for non-targeted metabolomics analysis.

### 4.3. Sample Extraction for Water Dropwort

The stems, leaves, and roots of water dropwort at the juvenile stage (0.1 g each) were ground, placed into 15-mL glass tubes, and frozen. Approximately 100 mg of frozen plant samples were treated with 2 mL of 4 M aqueous NaOH. The mixed solution was hydrolyzed at 40 °C for 2 h in a gas bath via shaking and with protection from light. The pH value was adjusted to 2 by adding 4 M aqueous HCl. The mixture was then shaken with 2 mL of n-hexane at room temperature at 4660 g for 20 min to remove the n-hexane layer. Ethyl acetate (2 × 2 mL) was used to extract the aqueous layer, and the mixed extracts were concentrated to nearly dry on a rotary evaporator at 35 °C under reduced pressure. The residue was dissolved in 200 μL of 50% methanol/water and transferred to insert-equipped vials for further targeted metabolomics analysis.

### 4.4. Metabolomics Determination of Allelochemicals

Analyses of culture water were carried out via ultra-high-performance liquid chromatography (Vanquish UPLC, Thermo Fisher, Waltham, MA, USA) and high-resolution mass spectrometry (Q Exactive™, Thermo Fisher). The LC-MS/MS system was operated under the following conditions: HPLC column, Waters HSS T3 (50 × 2.1 mm, 1.8 µm, Waters Corporation, Milford, MA, USA); column temperature, 40 °C; flow rate, 0.3 mL/min; injection volume, 2 μL; solvent system, water (0.1% acetic acid): acetonitrile (0.1% acetic acid); gradient program, 90:10 *v*/*v* at 0 min, 90:10 *v*/*v* at 1.0 min, 90:10 *v*/*v* at 7.0 min, 90:10 *v*/*v* at 7.1 min, 90:10 *v*/*v* at 9.0 min. The extraction of water dropwort was subjected to non-targeted analysis using a UPLC-Orbitrap-MS system (Vanquish UPLC, Thermo Fisher). The analytical conditions were as follows: UPLC column, Waters HSS T3 (1.7 μm, 2.1 × 50 mm, Waters Corporation); column temperature, 40 °C; flow rate, 0.3 mL/min; injection volume, 2 μL; solvent system, water (0.1% acetic acid): acetonitrile (0.1% acetic acid); gradient program, 90:10 *v*/*v* at 0 min, 90:10 *v*/*v* at 2.0 min, 40:60 *v*/*v* at 6.0 min, 40:60 *v*/*v* at 8.0 min, 5:95 *v*/*v* at 8.1 min, 95:5 *v*/*v* at 12.0 min.

HRMS data were recorded on a Q Exactive™ hybrid Q–Orbitrap mass spectrometer equipped with a heated ESI source (Thermo Fisher) utilizing the SIM MS acquisition methods. The ESI source parameters were set as follows: spray voltage, −2.8 kV/3.0 kV; sheath gas pressure, 40 arb; aux gas pressure, 10 arb; sweep gas pressure, 0 arb; capillary temperature, 320 °C; aux gas heater temperature, 350 °C.

### 4.5. Inhibitory Effect of the Allelochemicals on the Growth of M. aeruginosa

#### 4.5.1. Test 1: The Inhibitory Effect of the Water Dropwort Culture Water on the Growth of *M. aeruginosa*

Water dropwort samples in the juvenile stage, including the roots, were transplanted to the laboratory from an outdoor, large-scale, soilless planting field in Suqian, Jiangsu Province, China, in May 2020. First, the whole plants, including the roots, were rinsed with tap water and washed thrice with sterile water. Next, the whole plants with a fresh weight of 150 g were planted in purified water for one week. The culture water was then obtained after the removal of plant residues and microorganisms via filtering with three layers of sterile gauze and through a 0.22-μm filter. The 100-mL solution of *M. aeruginosa* in an exponential growth phase with a cell density of 10 × 10^6^ cells/mL, which was simultaneously cultured with BG-11 medium, was first transferred to sterile 250-mL Erlenmeyer flasks and adapted to grow for 2 days. Then, 0, 25, and 50 mL of prepared culture water was respectively added to Erlenmeyer flasks containing 100 mL of *M. aeruginosa*. The total volume of the culture solution in each group was maintained at 200 mL by replenishing sterile water, and the final cell concentration was 2 × 10^7^ cells/mL. All treatments included three parallel samples. Cultures without the culture water served as the control group. All cultures were maintained under the conditions of a temperature of 25 °C and a light-dark regime of 12 h: 12 h with 4000 lx of illumination. Each Erlenmeyer flask was shaken thrice per day, and the position was randomly changed to reduce the effect of light on algal growth.

#### 4.5.2. Test 2: The Inhibitory Effect of Phenolic Acids on the Growth of *M. aeruginosa*

The inhibitory mechanism of the growth of *M. aeruginosa* blooms via the allelochemicals in water dropwort was revealed by respectively adding ferulic, salicylic, and benzoic acids (Aladdin Reagents Co., Ltd., Shanghai, China). The concentration gradients of both salicylic acid and benzoic acid were set to 25, 30, 35, and 40 mg/L, and the concentration gradient of ferulic acid was set to 30, 40, 50, and 60 mg/L. Different concentrations of salicylic, benzoic, and ferulic acids were separately mixed with BG-11 medium as a stock solution of 500 mg/L, which was then autoclaved at 121 °C for 30 min. Then, 10 mL of *M. aeruginosa* in the logarithmic growth phase from the BG-11 growth medium was added to 500-mL sterile Erlenmeyer flasks containing different concentrations of phenolic acids, which caused the initial algal density of *M. aeruginosa* to be approximately 10^4^–10^5^ cells/mL. In the control group, the allelochemicals were replaced with the BG-11 medium, and each group had three parallel samples. All the flasks were cultured under the conditions of a temperature of 25 °C and a light-dark regime of 12 h: 12 h with 4000 lx of illumination in a laboratory incubator for 9 days, and the relevant indicators were regularly determined.

### 4.6. Cell Density and Chl-a

*M. aeruginosa* grew in a monolayer and the cells were evenly distributed in the culture solution; thus, the algal density was proportional to the optical density [78]. The cell density of *M. aeruginosa* was based on OD_680_, which was determined daily via spectrophotometry. The inhibition rate (IR) was determined by the following equation [69,79]:

IR(%) = (1 − N/N_0_) × 100%,

where N is the density of algae in the treatment group (expressed by OD_680_), and N_0_ is the density of algae in the control group (expressed by OD_680_) in the same period.

The chlorophyll a (Chl-a) concentration of *M. aeruginosa* was analyzed by the ethanol extraction method every 3 days [80,81]. The supernatant was determined using a UV-Vis spectrophotometer at 665 and 649 nm (UV-1600, Mapada Instruments, Shanghai, China) and ethanol as a control. The Chl-a concentration (mg/L) was determined by the following equation.

Chl-a = 13.9 × A665 − 5.76 × A649

### 4.7. Fluorescence Parameters F_v_/F_m_ and F_0_

The fluorescence parameters *F*_v_*/F*_m_ and *F*_0_ were determined by a Photon Systems Instrument (AquaPen AP110/P, Ecological Technology, Pleasanton, CA, USA) every 3 days. *F*_v_*/F*_m_ is the optimal/maximal photochemical efficiency of photosystem II (PSII), which indicates the intrinsic efficiency of PSII or the maximum light energy conversion efficiency of PSII determined after dark adaptation [82,83]. *F*_0_ is the initial fluorescence (minimal fluorescence) or the fluorescence output when the reaction center of PSII is fully opened of dark-adapted cells.

### 4.8. Antioxidant Enzyme Activities

The sample solution of *M. aeruginosa* was centrifuged for 10 min to collect cells. Then, 0.1 g of cells were broken with liquid nitrogen and re-suspended with phosphate buffer (pH = 7.0) at the temperature of 4 °C. The homogenate was centrifuged at 13,680× *g* for 10 min. The enzyme supernatant was preserved at −80 °C for further use. Superoxide dismutase (SOD) activity was determined according to the method of Beauchamp and Fridovich [84], catalase (CAT) activity was determined according to the method of Giannoplities and Ries [85], and peroxidase (POD) activity was determined according to the method of Evans [86].

### 4.9. Cell Morphology Assays

After cultivation, a certain amount of the sample was centrifuged at 1520× *g* for 10 min to collect algal cells, after which 2.5% (*v*/*v*) of glutaric dialdehyde solution prepared by phosphate buffer (pH = 7.0) was added to the fixed cells. The sample was stored in a refrigerator at 4 °C for further use. The samples were coated with gold at 15 mA for 110 s. The cell morphologies of *M. aeruginosa* were photographed using a scanning electron microscope (Quanta 200, Thermo Fisher) under high vacuum at 20 kV.

### 4.10. Data Processing and Statistical Analysis

The metabolomics data were acquired on the Q Exactive™ mass spectrometer using the Xcalibur 4.1 system (Thermo Fisher), and were then processed using Progenesis QI software (Waters Corporation, Milford, MA, USA). Quantified data were output into excel format. Statistical analysis was performed with Origin 2018 and SPSS 26.0 software for Windows. The data are expressed as the mean ± standard error. One-way analysis of variance (ANOVA) was used to determine the differences among different phenolic acids, and differences were considered significant at *p* < 0.05. The figures were constructed with R (pheatmap package), Origin 2018, and Microsoft Office software.

## 5. Conclusions

In this study, water dropwort culture water was first found to inhibit the growth of *M. aeruginosa*. Three hundred and six metabolites were identified in the culture water, among which 33 have been proven to have a potential allelopathic function. In addition, there were 15 phenolic acids among these 33 allelochemical compounds, which was much more than the fatty acids (nine types) and terpenes (nine types). Thus, the culture water with water dropwort at different development stages was not only rich in allelochemicals, but was also particularly abundant in phenolic acids at the juvenile stage. Regarding water dropwort itself, 18 phenolic acids were first discovered in all the organs of the water dropwort by targeted metabolomics analysis; the phenolic acids were found to be mainly synthesized in the leaves and then transported to the roots, and were ultimately released into the culture water for the inhibition of the growth of *M. aeruginosa*. Then, three types of phenolic acids synthesized in water dropwort, i.e., benzoic, salicylic, and ferulic acids, were selected to clarify their inhibitory effects on the growth of *M. aeruginosa* and their mechanisms. The inhibitory effects were found to be concentration- or dose-dependent, although the initial inhibitory concentrations of the three phenolic acids to the algal cells were different. The highest inhibition rates were respectively found to be 92.86% (for 40 mg/L benzoic acid), 88.03% (40 mg/L salicylic acid), and 75.14% (60 mg/L ferulic acid). Via SEM observation, it was confirmed that the algal cells were more sensitive to benzoic acid than to salicylic and ferulic acids, and all three phenolic acids caused the distinct alteration of the *M. aeruginosa* cell morphology, reduced the number of cells, damaged the cell membrane, and caused the leakage of the cell contents, resulting in the inhibition of algal cell growth. High concentrations of phenolic acids can significantly reduce the content of Chl-a, decrease the values of *F*_0_ and *F*_v_*/F*_m_, and increase the antioxidant enzyme activity of SOD, POD, and CAT. Therefore, the simultaneous effects on cell division, the cell membrane integrity, Chl-a synthesis, PSII efficiency, and the antioxidant enzyme systems of *M. aeruginosa* are the main mechanisms by which phenolic acids inhibit algal growth. In comprehensive consideration of these results, both culture water with rich allelochemicals and biological algae inhibitors made from water dropwort could be used to control the growth of noxious algae in the future. This study provides a reference for the dual goals of the resource utilization of water dropwort after bioremediation and the development of an environmentally friendly, low-cost, and effective noxious algae control agent.

## Figures and Tables

**Table 1:** The formula of the BG-11 medium for *M. aeruginosa*.

Component	Stock Solution (g/L)	Component	Stock Solution (g/L)
NaNO_3_	1.5	Na_2_CO_3_	0.02
K_2_HPO_4_·3H_2_O	0.04	H_3_BO_4_	0.00286
MgSO_4_·7H_2_O	0.075	MnCl_2_·H_2_O	0.00181
CaCl·2H_2_O	0.036	ZnSO_4_·7H_2_O	0.000222
Citric acid	0.006	CuSO_4_·5H_2_O	0.000079
Ferric ammonium citrate	0.006	Na_2_MoO_4_·2H_2_O	0.00039
EDTA	0.001	Co(NO_3_)_2_·6H_2_O	0.000049

## Data Availability

All the data supporting this article were included in the main text.

## Supplementary Materials

The following are available online at https://www.mdpi.com/article/10.3390/plants10122653/s1, Figure S1: Effects of different concentrations of ferulic acid, salicylic acid and benzoic acid, respectively, on the growth of *M. aeruginos*. The data were reported as mean ± standard error, Table S1: Targeted metabolomics analysis for the phenolic acids in the whole plant of water dropwort at juvenile stage.
