# Supplementary material for: Abundant Allelochemicals and the Inhibitory Mechanism of the Phenolic Acids in Water Dropwort for the Control of Microcystis aeruginosa Blooms
Source: Plants (Basel). 2021 Dec 2;10(12):2653. doi: 10.3390/plants10122653 (PMC8707890; doi:10.3390/plants10122653)
Supplement: Supplementary file 1 [file plants-10-02653-s001.zip › plants-1485724-supplementary.pdf]

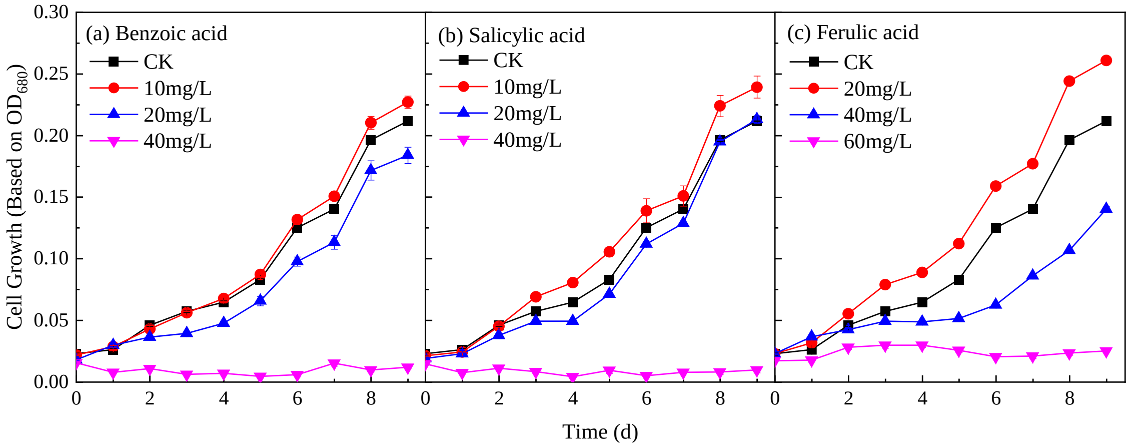

(A) Change of the cell density for the *M. aeruginosa* with different concentrations of phenolic acids.

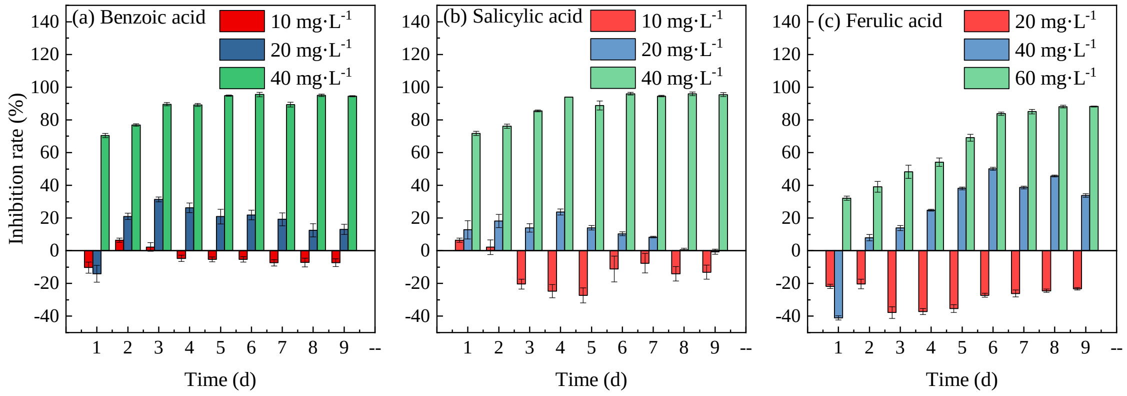

(B) Inhibition rate of the *M. aeruginosa* with different concentrations of phenolic acids.

Figure S1. Effects of different concentrations of ferulic acid, salicylic acid and benzoic acid, respectively, on the growth of *M. aeruginos*.The data were reported as mean ±standard deviation.

Table S1. Targeted metabolomics analysis for the phenolic acids in the whole plant of water dropwort at juvenile stage

Chemicals (μg·g^{-1}, FW)	Root	Stem	Leaf
Gallic acid	0.3200 ± 0.0010b	0.2553 ± 0.1058b	3.2610 ± 0.0090a
Vanillic acid	0.713 ± 0.0090b	0.5150 ± 0.0480b	4.5228 ± 0.3128a
Caffeic acid	7.7050 ± 0.5230b	2.9210 ± 0.1210c	37.2612 ± 1.5658a
4-Hydroxycinnamic acid	3.8575 ± 0.2045a	1.7450 ± 0.6495a	3.8125 ± 0.2305a
Ferulic acid	11.0535 ± 0.4375b	8.1765 ± 0.2365b	15.1996 ± 1.1453a
Protocatechualdehyde	0.2850 ± 0.0250b	0.1230 ± 0.0030b	0.8722 ± 0.0562a
4-Hydroxybenzoic acid	1.3393 ± 0.2315a	0.7445 ± 0.0055a	1.2471 ± 0.1164a
Vanillin	1.3397 ± 0.2295a	0.7375 ± 0.0255a	1.2886 ± 0.1712a
Syringaldehyde	0.1527 ± 0.0199b	0.1420 ± 0.0484b	1.2763 ± 0.0868a
roxy-3,5-dimethoxycinnamic acid	0.0600 ± 0.0040b	0.0985 ± 0.0135b	0.9310 ± 0.1520a
Phe(L-Phenylalanine)	0.0185 ± 0.0055b	0.0163 ± 0.0023b	0.0679 ± 0.0089a
3,4-Dihydroxybenzoic acid	0.0670 ± 0.0080b	0.0797 ± 0.031b	0.1653 ± 0.0148a
Syringic acid	0.0675 ± 0.0095a	0.0183 ± 0.0030b	0.1034 ± 0.0034a
Benzoic acid	0.1390 ± 0.0169a	0.0867 ± 0.0148b	0.1407 ± 0.0082a
Trans-Cinnamic acid	0.0153 ± 0.0029b	0.0185 ± 0.0005b	0.1175 ± 0.0165a
L-Epicatechin	0.0017 ± 0.0003a	0.0003 ± 0.0003b	0.0015 ± 0.0003a
Salicylic acid	0.0210 ± 0.0068b	0.0130 ± 0.0038b	0.0453±0.00852a
Hydrocinnamic acid	0.04750 ± 0.0015a	0.0017 ± 0.0003c	0.0116±0.0003b

Note: Lowercase letters indicate the significant difference of the same phenolic acid in different organs. Plus and minus signs indicate standard deviation.
